# Anxiety disorders and age-related changes in physiology

**DOI:** 10.1192/j.eurpsy.2022.487

**Published:** 2022-09-01

**Authors:** J. Mutz, T. Hoppen, C. Fabbri, C. Lewis

**Affiliations:** 1 King’s College London, Social, Genetic And Developmental Psychiatry, London, United Kingdom; 2 University of Münster, Institute Of Psychology, Münster, Germany; 3 University of Bologna, Department Of Biomedical And Neuromotor Sciences, Bologna, Italy

**Keywords:** Anxiety, Ageing, Physiology, UK Biobank

## Abstract

**Introduction:**

Anxiety disorders are leading contributors to the global disease burden, highly prevalent across the lifespan, and associated with substantially increased morbidity and early mortality.

**Objectives:**

The aim of this study was to examine age-related changes across a wide range of physiological measures in middle-aged and older adults with a lifetime history of anxiety disorders compared to healthy controls.

**Methods:**

The UK Biobank study recruited >500,000 adults, aged 37-73, between 2006-2010. We used generalised additive models to estimate non-linear associations between age and hand-grip strength, cardiovascular function, body composition, lung function and heel bone mineral density in cases vs. controls.

**Results:**

The main dataset included 332,078 adults (mean age = 56.37 years; 52.65% females). In both sexes, individuals with anxiety disorders had lower hand-grip strength and blood pressure than healthy controls, while their pulse rate and body composition measures were higher. Case-control differences were larger when considering individuals with chronic and/or severe anxiety disorders, and differences in body composition were modulated by depression comorbidity status. Differences in age-related physiological changes between female anxiety disorder cases and healthy controls were most evident for blood pressure, pulse rate and body composition, while in males for hand-grip strength, blood pressure and body composition. Most differences in physiological measures between cases and controls tended to decrease with age increase.

**Conclusions:**

Individuals with a lifetime history of anxiety disorders differed from healthy controls across multiple physiological measures, with some evidence of case-control differences by age. The differences observed varied by chronicity/severity and depression comorbidity.

**Disclosure:**

JM receives studentship funding from the Biotechnology and Biological Sciences Research Council (BBSRC) and Eli Lilly and Company Limited. CML is a member of the Scientific Advisory Board of Myriad Neuroscience. CF and THH declare no relevant conflict of

